# Parasomnias during trans‐meridian and long‐distance travel: Critical literature review and clinical practice recommendations

**DOI:** 10.1111/jsr.13672

**Published:** 2022-06-20

**Authors:** Sinead M. Walsh, Cameron L. Forward, Gerard T. Flaherty

**Affiliations:** ^1^ School of Medicine National University of Ireland Galway Galway Ireland; ^2^ Department of Respiratory and Sleep Medicine Galway University Hospitals Galway Ireland; ^3^ School of Medicine International Medical University Kuala Lumpur Malaysia

**Keywords:** management, parasomnias, pre‐travel health consultation, trans‐meridian travel, triggers

## Abstract

Parasomnias are undesirable events that occur during sleep. They can be classified into rapid eye movement parasomnias and non‐rapid eye movement parasomnias. Those who experience parasomnias may be anxious about travel for many reasons, including the occurrence of unwanted events during the trip, increased exposure to environmental trigger factors, and the propensity for harm to occur due to unfamiliar surroundings while travelling. There is a paucity of literature examining this area. This review summarizes the relevant literature and the clinical experience of the authors to compile clinical practice recommendations. The clinical features of parasomnias and how they relate to trans‐meridian and long‐distance travel are described. Triggers for non‐rapid eye movement parasomnias, particularly the use of sedative hypnotic drugs, alcohol, drug withdrawal, sleep deprivation, emotional stress and environmental stimulations, are described. Management of parasomnias whilst travelling is reviewed, with a particular focus on trigger minimalization. The role for clonazepam and melatonin is outlined. At the pre‐travel health consultation, the physician is strongly advised to screen the traveller for co‐morbid sleep conditions, which exacerbate parasomnias. Areas for further research are explored, including the extent to which these sleep disorders impact on the travel experience.

## 1 INTRODUCTION

Parasomnia derives from the Greek word “*para*” (meaning “alongside of”) and the Latin word “*somnus*” (meaning “sleep”), describing events that accompany sleep. Parasomnias are undesirable behavioural, autonomic and experiential events that occur during the entry to sleep, during any stage of sleep, or during partial or full arousals from any stage of sleep (Sateia, 2014). They can be classified according to the sleep stage during which they occur into rapid eye movement (REM) parasomnias, non‐REM (NREM) parasomnias and other parasomnias. REM parasomnias encompass REM sleep behaviour disorder (RBD), nightmare disorder, and recurrent isolated sleep paralysis. NREM parasomnias comprise disorders of arousal (namely sleep walking, sleep terror, confusional arousals), and the lesser understood entities of sleep‐related sexual behaviours (sexsomnia) and sleep‐related eating disorder (SRED). Other parasomnias include catathrenia, sleep enuresis and exploding head syndrome (Sateia, 2014).

The prevalence of RBD is estimated at approximately 1.15% in adults over 60 years old (Kang et al., 2013). Two percent–6% of adults report frequent (weekly) nightmares (Levin & Nielsen, 2007). Up to 20% of children report frequent nightmares (Gauchat, Séguin, & Zadra, 2014). NREM parasomnia prevalence varies with age. Disorders of arousal are more common in childhood and decrease with increasing age (Hrozanova, Morrison, & Riha, 2019). Children have a high prevalence of sleep terrors (39.8%) and sleep walking (14.5%; Petit et al., 2007). In adults, sleep terror prevalence drops to 2.7%, whilst sleep walking affects 1.7% (Bjorvatn, Grønli, & Pallesen, 2010). A substantial proportion of patients with a parasomnia are likely to engage in trans‐meridian and long‐distance travel, for work or leisure purposes. Travellers who experience parasomnias may be apprehensive about travel for many reasons, including the occurrence of unwanted events during the trip, increased exposure to environmental trigger factors, and the propensity for harm to occur due to unfamiliar surroundings whilst travelling. There is a paucity of guidance in the literature in relation to the issues surrounding trans‐meridian and long‐distance travel in individuals with a parasomnia. This review of the topic was generated from the available literature and the clinical experience of the authors, and to the best of our knowledge it is the first attempt to consider parasomnias in the context of travel.

## 2 METHODS

The PubMed/MEDLINE database and Google Scholar were accessed through January 2022 to source relevant literature. Combinations of the following keywords were used as search terms: “travel”, “international travel”, “travel advice” and “NREM parasomnia”, “REM parasomnia”, “sexsomnia”, “sleep related eating disorder”. Articles were restricted to those published in English. References of identified articles were screened for additional sources.

### 2.1 Clinical features of parasomnias

The NREM parasomnias typically result from incomplete arousal from slow‐wave sleep (Castelnovo, Lopez, Proserpio, Nobili, & Dauvilliers, 2018). Sleep walking, sleep terror and confusional arousal occur when there is an incomplete dissociation of NREM sleep into the wakefulness state. This leads to clinical states of sleep with central nervous system activation of skeletal muscle and autonomic networks. Those affected describe recurrent episodes of incomplete awakening from sleep. The individual appears confused and disoriented for several minutes after the episode. There is an inappropriate or absent responsiveness to the efforts of others to intervene or redirect the individual during an episode. Afterwards, there is limited or no associated cognition or dream imagery, and the affected individual describes partial or complete amnesia for the episode (Castelnovo et al., 2018). Trauma associated with sleep walking is rare but can be potentially life‐threatening. Cases of paraplegia, severe head injury, concussion and bone fractures have all been reported (Sauter et al., 2016). Nocturnal sleep walking also causes daytime sleepiness (Montplaisir, Petit, Pilon, Mongrain, & Zadra, 2011). This can have implications for travellers who plan on engaging in adventure sports, or possibly make them more vulnerable to physical assault.

The RBD occurs when dream enactment or disruptive behaviours emerge during REM sleep, accompanied by loss of normal REM sleep muscle atonia on polysomnography (PSG). RBD is highly variable across nights and among patients, in terms of event frequency, duration and behaviour type (Dauvilliers et al., 2018). During these events, abnormal behaviours emerge during REM sleep, having the potential to cause significant injury or sleep disruption such as talking, shouting, gesturing, grabbing, flailing of arms, punching, kicking, sitting up or jumping out of bed (Schenck & Mahowald, 2002). The screams and swearing can awaken the individual, their bed partner and others in the building. This can be a particular source of embarrassment, especially when individuals are sleeping outside their usual home environment, as occurs whilst travelling (Dauvilliers et al., 2018). Shared dormitory or hostel accommodation during international travel present situations where this behaviour may be particularly disruptive or even invite aggression from often unfamiliar room‐mates. Once awake, individuals are alert and can recall the dream content. RBD is strongly associated with the development of α‐synucleinopathies. These are neurodegenerative disorders characterized by the accumulation of α‐synuclein in neurons, nerve fibres or glial cells. The group includes Parkinson's disease, Lewy body dementia and multiple system atrophy. The medical fitness to fly concerns surrounding older air travellers with cognitive impairment have been discussed elsewhere (Sadlon, Ensslin, Freystätter, Gagesch, & Bischoff‐Ferrari, 2021).

Nightmare disorder is another REM parasomnia. It is characterized by disturbing, internally generated conscious experiences that usually awaken the sleeper from REM sleep. Common accompanying emotions include anxiety and fear, with a corresponding somatic response of tachycardia, tachypnoea and diaphoresis (Morgenthaler et al., 2018). The individual has immediate recall and full arousal upon wakening. This can be a source of anxiety for the traveller who is aroused from a nightmare in an unfamiliar sleep setting such as on board an aircraft, overnight sleeper train or hotel room.

### 2.2 Triggers for NREM parasomnias

A genetic propensity to develop the NREM disorders of arousal is recognized in up to 60% of cases, although the mode of transmission is unclear (Petit et al., 2015). An individual with a genetic predisposition can encounter environmental triggers that precipitate NREM parasomnias (Pressman, 2007). Many of the environmental triggers for NREM parasomnias are exacerbated by trans‐meridian and long‐distance travel. Use of sedative hypnotic drugs (e.g. zolpidem and zaleplon), occasionally prescribed for the short‐term treatment of flight phobia or insomnia during travel, have been associated with the emergence or worsening of NREM parasomnias (Ferentinos & Paparrigopoulos, 2009). Reports in main‐stream media regularly describe weary flight attendants dealing with so‐called “*ambien zombies*”. Passengers with severe flight phobia may not always comply with pre‐travel advice to avoid consuming alcohol in conjunction with the use of sedative hypnotic agents during flight, leading to the potential for over‐sedation, paradoxical disinhibition or flight parasomnias. Other pharmacological risk triggers include lithium, quetiapine, olanzapine and fluoroquinolones (Howell, 2012), the latter often prescribed for self‐treatment of moderate to severe travellers' diarrhoea in travellers to countries with a high risk for food‐ and water‐borne disease (Riddle et al., 2017). Mefloquine, previously commonly used for prophylaxis and treatment of *Plasmodium falciparum* malaria, was associated with recurring nightmares in 59% of those who consumed it (Ringqvist, Bech, Glenthøj, & Petersen, 2015). Use of this medication has decreased dramatically in recent years due to its high rate of neuropsychiatric adverse effects.

Trans‐meridian and long‐distance travel is often associated with reduced opportunities for sleep and sleep deprivation. The “first‐night effect” describes the common event of disturbed sleep in a novel environment. When sleeping in a new environment, one brain hemisphere has been shown to be more vigilant, with regional asymmetrical slow‐wave activity noted on PSG recordings (Tamaki, Bang, Watanabe, & Sasaki, 2016). Overnight air, rail or bus travel, flight delays or cancellations necessitating attempts at sleep in noisy airports, and sharing of accommodation particularly among backpackers or trekkers are examples of the types of sleep disruption associated with travel overseas. The recovery from sleep deprivation and associated increased deep or slow‐wave sleep can exacerbate NREM parasomnias (Zadra, Pilon, & Montplaisir, 2008). Situational stress, emotional stimulation prior to sleep onset and environmental noises are frequently experienced by travellers in new environments. These can induce arousals into wakefulness from the normal sleep process and trigger NREM parasomnias (Hrozanova et al., 2019). Sleep deprivation for over 30 hr and travelling across time zones were believed to have led to an individual sleep‐walking out of a second‐storey window and sustaining serious bodily injuries (Gunn & Gunn, 2020). Somniloquy, abnormal connected speech during sleepwalking, was described in an adolescent boy after a 13‐hr flight (Hurwitz & Richardson, 2010). The associated sleep loss, stress and jet lag were recognized accompanying trigger factors.

Triggers for REM parasomnias are less well described. The use of tricyclic anti‐depressants and selective serotonin reuptake inhibitors have occasionally been associated with worsening of RBD; however, clear temporal relationships have not been established (Olson, Boeve, & Silber, 2000). Alcohol intake or acute withdrawal from alcohol can also trigger or aggravate RBD (Dauvilliers et al., 2018). Passengers on aircraft may consume alcohol in an effort to make the journey more enjoyable and tolerable (Girasek & Olsen, 2009). Those with RBD need to be cautioned about the adverse effect this may have on their condition.

Drug tourism is the phenomenon by which a person travels to a particular location due to the easy access of licit or illicit drugs and related services (Valdez & Sifaneck, 1997). Use of illicit recreational drugs, particularly cocaine and amphetamines, places drug tourists at risk of unintentional injury and interpersonal violence (Flaherty, Maxemous, Nossier, & Bui, 2017). These drugs suppress REM sleep. Withdrawal from cocaine and amphetamines causes a REM sleep rebound, and onset and exacerbation of RBD (Schenck, Hurwitz, & Mahowald, 1993a). The dream content associated with RBD and cocaine misuse is predominantly that of drug cravings and consumption (Jiménez‐Correa et al., 2022).

### 2.3 Management of parasomnias during travel

The cornerstone of management of NREM parasomnias whilst travelling is the avoidance of triggers, practising healthy sleep routines, and ensuring a safe sleeping environment. Sleep hygiene refers to habits and practices that are conducive to sleeping well on a regular basis. Travellers should be advised to maintain a regular bedtime and wake‐up time. This can be difficult to achieve when individuals are travelling across multiple time zones and temporary disturbances occur to their circadian rhythm (jet lag). The misalignment between the timing of the sleep–wake cycles generated by the endogenous circadian clock and that required in the new time zone can trigger parasomnias if not effectively managed. Any high‐intensity exercise should be performed earlier in the day. Heavy meals should not be consumed prior to bedtime. Screen time prior to sleep should be limited and the bedroom should be kept dark. Use of opaque eye shades helps to facilitate this. The ambient room temperature should be maintained between 18°C and 20°C. Extraneous noise should be minimized, but this is not always easily accomplished in hotel or hostel accommodation, and the advantages of using noise‐reducing ear plugs must be balanced against the potential hazard of failing to respond to a fire alarm while sleeping (Flaherty, Hession, & Cuggy, 2016).

It is essential that the sleep environment is safe for travellers with a parasomnia. All potentially dangerous items should be removed from the bedroom, including nightstands, lamps or other objects that could cause injury. Windows should be locked and consideration could be given to use of door alarms, especially for sleep‐walkers. If an individual is travelling accompanied, the sleeping partner should be advised to provide quiet guidance to get the sleep‐walker back into bed (Harris & Grunstein, 2009). In the case of a history of self‐injurious behaviours, consideration should be given to requesting sleeping accommodation on the lowest floor of the building and using a mattress on the floor.

“Sleep driving” is a variant of NREM parasomnias, and describes individuals who arouse from deep sleep, leave their beds, enter their cars and drive, often in an unsafe manner (Pressman, 2011). It also occurs with improper use of sedatives, in particular the z‐drugs, zolpidem and zopiclone (Southworth, Kortepeter, & Hughes, 2008). Such an individual should be advised not to have access to a motor vehicle whilst engaging in international travel.

Sexsomnia is a NREM parasomnia that involves any sexual behaviour carried out whilst the person is asleep. Sexsomnia is associated with widespread autonomic activation including erection and ejaculation in males, and vaginal lubrification in females. The clinical features vary and can include any or all of explicit vocalizations, masturbation, sexual fondling or sexual intercourse (Béjot et al., 2010). Nocturnal PSG recordings in those with sexsomnia demonstrate abrupt and spontaneous arousals from slow‐wave sleep. Precipitating factors identified by Schenck et al. are all exacerbated by travel, including sleep deprivation, stress, fatigue, alcohol consumption and drug misuse (Schenck, Arnulf, & Mahowald, 2007). There has been a marked increase in the number of inflight sexual assaults reported in recent years (Deliv & Flaherty, 2020). The reasons for this are multifactorial and include sexsomnia. Travellers with sexsomnia should take every precaution to avoid triggers. Shared dormitory or hostel settings should be avoided to reduce the occurrence of unwanted events that may cause significant psychological distress to all involved, and present a risk of litigation and incarceration for the traveller.

The SRED describes recurrent episodes of dysfunctional eating that occur after an arousal from sleep during the main sleep period (Schenck, Hurwitz, Bundlie, & Mahowald, 1991; Schenck, Hurwitz, O'Connor, & Mahowald, 1993b). This is a female‐predominant NREM parasomnia that is not hunger driven. Affected individuals gain weight due to nightly eating of high‐calorie “comfort” foods. Inappropriate consumption of food or non‐nutritive substances occurs, with bizarre food choices, and occasionally inedible or toxic items can be ingested, such as raw meat, pet food or household cleaning products (Inoue, 2015). Affected individuals have varying levels of consciousness during SRED episodes, ranging from partial consciousness to total unawareness. SRED is associated with ingestion of sedative hypnotic medications and other sleep disorders (Inoue, 2015). Travellers who experience SRED should have non‐poisonous food substances nearby in case they experience an episode of dysfunctional eating. They should also try to ensure that they do not have access to unfamiliar cooking utensils, which may increase the risk of injuries such as burns or lacerations.

### 2.4 Pharmacological interventions for parasomnias when travelling

If parasomniac behaviours persist despite control of exacerbating factors, removal of triggers and treatment of co‐morbidities, pharmacological interventions may be considered. However, the evidence for pharmacological therapy is limited and high‐quality evidence is lacking. Clonazepam is a long‐acting benzodiazepine that has demonstrated efficacy in the treatment of injurious parasomnias (Schenck & Mahowald, 1996). Low‐dose clonazepam (0.5–1 mg daily) has been shown to improve features of NREM disorders of arousal (Drakatos et al., 2019; Schenck & Mahowald, 1996). Nightly administration of clonazepam also improves RBD symptoms, with patients demonstrating improvement in dream enacting behaviours for years or decades (Schenck & Mahowald, 1996). However, the abrupt withdrawal of clonazepam can result in a rebound of RBD symptoms (Dauvilliers et al., 2018). A patient who is prescribed clonazepam for long‐term use should not forget to bring their treatment whilst travelling, and should continue to take the drug as prescribed. Patients should be counselled about the side‐effects of clonazepam, including dizziness, daytime sleepiness, impaired gait and altered cognition. We recommend that a person with a parasomnia should never take hypnotic medication for the first time whilst travelling, due to the possibility of the hypnotic leading to the worsening of NREM parasomnias (Ferentinos & Paparrigopoulos, 2009), and possible medico‐legal forensic consequences. If a hypnotic medication is deemed necessary for travel, the traveller should take it for at least several nights before travelling to ensure there are no adverse effects.

Administration of melatonin in patients with RBD has demonstrated efficacy (Kunz & Bes, 1999). Melatonin can be commenced at a low dose for 5–7 days, and then gradually increased every 5–7 days to a maximum dose of 12 mg at night (Dauvilliers et al., 2018). In patients with concomitant RBD and obstructive sleep apnea syndrome (OSAS), the apnea‐related arousals are known to provoke RBD‐like symptoms and co‐administration of melatonin can lead to improved RBD symptoms (Schaefer, Kunz, & Bes, 2017). Timed melatonin administration is also indicated to reduce symptoms of jet lag and improve sleep following trans‐meridian and long‐distance travel (Morgenthaler et al., 2007). Melatonin is not universally available and, while it is available as a food supplement in pharmacies in many countries, some jurisdictions restrict its sale. It may not be possible to guarantee the purity of commercially available melatonin preparations marketed as food supplements online.

Cognitive behavioural therapy (CBT) has also been used in the treatment of parasomnias, despite the lack of high‐quality evidence. Types of CBT include stress reduction strategies, hypnotherapy, relaxation therapy, and anticipatory or scheduled awakening (Galbiati, Rinaldi, Giora, Ferini‐Strambi, & Marelli, 2015). Imagery rehearsal therapy is also useful in nightmare disorder. These should be discussed with the traveller as possible interventions that may improve their sleep experience overseas. CBT therapy is available in person or online.

### 2.5 Pre‐travel health consultation

The pre‐travel health consultation provides an opportunity for the clinician to discuss general specific travel health precautions. If not already performed, an individual with a parasomnia should be screened for co‐morbid sleep disorders. Conditions such as periodic limb movements in sleep (PLMS) and OSAS induce disruptions of the normal sleep processes and can exacerbate NREM parasomnias. Satisfactory treatment of PLMS and OSAS can lead to improved control of parasomnia symptoms, irrespective of NREM‐parasomnia phenotype and underlying PLMS or OSAS severity (Drakatos et al., 2019). In RBD, co‐morbid OSAS should also be adequately treated with continuous positive airway pressure (CPAP) to improve RBD‐like behaviours and to allow clonazepam prescription, which can worsen OSAS symptoms (Dauvilliers et al., 2018). If a patient has co‐existing OSAS requiring CPAP use, it is strongly advised that patients planning on travelling with CPAP prepare adequately in advance (Walsh & Flaherty, 2020). Travellers should ensure to bring their CPAP machine or mandibular advancement device (MAD) with them to avoid a relapse of OSA as a trigger for NREM parasomnia while travelling. In addition, if a patient is taking medications for restless legs syndrome (RLS) or PLMS, they should be advised to bring these medications whilst travelling and to ensure ongoing compliance. All medications should be kept in their labelled prescription packaging, rather than being placed into an anonymous container, to prevent confiscation by customs officials and possible penalties. If the traveller is bringing medications used to treat parasomnias, these should be safeguarded. Other factors that induce arousals into wakefulness from the normal sleep processes and can exacerbate NREM parasomnias are a full bladder and pain. These symptoms should be enquired about and adequately treated if present. Practical advice for long‐haul air travel include limiting alcohol intake and avoiding pharmaceutically induced sleep. Travellers should be advised not to consume any alcohol within several hours of taking a hypnotic medication. A sleep kit should be packed, which includes an eye mask, ear plugs, travel pillow, light blanket, audiobook, headphones, relaxing music playlist, and water. It is recommended that the traveller inform their travel insurance provider about their pre‐existing parasomnia. Pre‐existing medical conditions are not usually covered under a general travel insurance policy. A traveller will typically need to contact their travel insurance provider about their parasomnia and purchase an add‐on to their policy (see Table 1 for clinical practice recommendations for travellers with parasomnias).

**TABLE 1:** Clinical practice recommendations for travellers with parasomnias

Avoid triggering substances	
	Avoid sedative hypnotic medications (can worsen NREM parasomnias)
	Minimize alcohol consumption while travelling
	Avoid recreational drugs
	Avoid other pharmacological triggers, especially fluoroquinolones (often prescribed for treatment of moderate to severe travellers' diarrhoea)
Minimize sleep deprivation	
	Minimize overnight travel where possible
	When backpacking, spend more than 1 night in locations to minimize the “first‐night effect” on sleep
	Minimize jet lag by limiting the number of time zones crossed in one journey
Avoid sharing accommodation	
	In those with sexsomnia, sharing a room in a hostel with others should be avoided to minimize the risk of alleged sexual assaults and adverse outcomes
Minimize situational stress	
	Plan journey meticulously in advance to minimize stress
Ensure a safe sleeping environment	
	Request sleeping accommodation on the lowest floor of the building
	Remove all dangerous objects from the bedroom
	If SRED, remove all potentially toxic substances that could be ingested or unfamiliar cooking utensils that can cause serious injury
	Lock all windows/sleep away from windows
	Consider door alarms
	If travelling accompanied, alert travel partner to parasomnia and provide guidance about calmly bringing the affected individual back to bed if parasomnia behaviours occur
Maintain meticulous sleep hygiene	
	Where possible, maintain consistent sleep–wake schedule
	Limit exposure to screens and bright lights
	Limit caffeine intake prior to sleep
	Avoid heavy meals prior to bedtime
	Avoid strenuous exercise close to bedtime
Ensure co‐morbid sleep disorder is adequately treated	
	For patients with OSA, bring treatment device (CPAP or MAD)
	For patients with RLS, bring RLS medications
Pharmacological therapy when indicated	
	Nightly low‐dose clonazepam (0.5–1 mg) improves features of NREM disorders of arousal and improves RBD symptoms
	Melatonin improves RBD symptoms
	Melatonin reduces symptoms of jet lag and improves sleep following travel across multiple time zones
CBT therapies (in‐person or online)	
	Stress reduction strategies, hypnotherapy, relaxation therapy, and anticipatory or scheduled awakening can be useful
	Imagery rehearsal therapy for nightmare disorder
Pre‐travel health consultation	
	Screen for co‐morbid sleep disorders and treat if detected
	Nocturia and nocturnal pain should be treated if present
	Provide practical advice for long‐haul air travel
	Pack a sleep kit
	Keep all medications in their labelled prescription bottles
	Safeguard medications used to treat parasomnias
	Advise patients not to consume any alcohol within several hours of taking a hypnotic medication
	Inform travel insurance provider of a pre‐existing parasomnia prior to travel

Abbreviations: CBT, cognitive behavioural therapy; CPAP, continuous positive airway pressure; MAD, mandibular advancement device; NREM, non‐rapid eye movement; OSA, obstructive sleep apnea; RBD, REM sleep behaviour disorder; RLS, restless legs syndrome; SRED, sleep‐related eating disorder.

### 2.6 Limitations and areas for further research

Although the understanding of parasomnias has greatly improved in recent years, much of the published research surrounding parasomnias is derived from case reports and case series. High‐quality evidence is therefore lacking. There are no large randomized controlled trials examining the management of parasomnias. There is a paucity of literature surrounding parasomnias and international travel. The small number of case reports that are published are included in this review. The effects of high altitude and hypoxaemia on parasomnias remain unexplored. While sleep at higher altitude is characterized by increased time spent awake, reduced slow‐wave sleep and reduced REM (Bloch, Buenzli, Latshang, & Ulrich, 2015), the implications at altitude or returning from altitude for those with a parasomnia are not known. The effect of periodic breathing leading to unrefreshed sleep and the association, if any, with parasomnias remains unexplored. Finally, the forensic consequences of parasomnias in travellers engaging in trans‐meridian and long‐distance travel remain unexplored and merit research.

## 3 CONCLUSION

In summary, parasomnias can place the traveller and those around them at risk of physical injury given the absence of conscious control and awareness during the unwanted events. First‐line interventions include treatment of co‐morbidities and exacerbating factors prior to travel, avoidance or minimization of triggers during travel, and ensuring safety of travellers in their new environment. Pharmacological therapy may be indicated if the parasomnias persist. Future research should shed light on the prevalence of parasomnias in international travellers and on the extent to which these sleep disorders impact on the travel experience.

## Data Availability

This is a review article.
